# Esophageal atresia: Factors influencing survival - Experience at an Indian tertiary centre

**DOI:** 10.4103/0971-9261.42564

**Published:** 2008

**Authors:** R. K. Tandon, Satendra Sharma, Shandip K. Sinha, Kumar Abdul Rashid, Ravi Dube, S. N. Kureel, Ashish Wakhlu, J. D. Rawat

**Affiliations:** Department of Pediatric Surgery and Neonatology, King George's Medical University, Lucknow -226 003, India

**Keywords:** Associated anomalies, esophageal atresia, prognosis, tracheoesophageal fistula

## Abstract

**Objective:**

To study the clinical profile of the cases of esophageal atresia (EA) and/or tracheoesophageal fistula (TEF) and various factors affecting the surgical and early postoperative management and their outcome.

**Materials and Methods:**

A prospective analysis of 127 cases of EA from February 2004 to May 2006 was performed. Waterston prognostic criteria were used for grading.

**Results:**

EA with TEF was the commonest type in 117 cases (92%). Associated congenital anomalies were present in 52 (41%) patients, the commonest being the cardiac anomalies, which was followed by the gastrointestinal anomalies. VACTERL was found in 6 (5%) cases. Prematurity, associated congenital anomalies, gap between esophageal ends and preoperative respiratory status were the significant factors affecting the survival (*P* = < 0.001). Primary extrapleural repair was the surgical approach in most of the patients. Azygos vein was preserved in 46 cases and no retropleural drainage was used in 27 cases. Staged procedures were performed in 19 cases, including 6 cases of isolated esophageal atresia. Pneumonitis and sepsis were the most common early postoperative complications (42%). Hypoxia and cardiorespiratory arrest were the most common causes of mortality (11 cases). Anastomotic leak complicated 13 cases, including 9 major and 4 minor leaks. Major leak followed by sepsis caused 7 deaths. Survival as per Waterston criteria was 100% in group A, 83% in group B and 22% in group C.

**Conclusion:**

Factors affecting the survival are major or life-threatening associated anomalies, long gap, pneumonia and sepsis at presentation or that acquired during hospitalization and major leaks. The high incidence of low birth weight, delayed diagnosis, poor referral, low-socio economic status and lack of advanced neonatological back up are important contributory factors to poor outcome.

## INTRODUCTION

Thomas Gibson[1] is credited with the first description of esophageal atresia (EA) with tracheoesophageal fistula (TEF) in 1697. Haight and Towsley[2] reported the first survivor following a primary anastomosis. After this, there was an improvement in the outcome of patients with EA/TEF, but the survival rate continued to be poor. Recently, significant advances have been made in the management of neonates with EA. This has resulted in a progressive decrease in mortality as a result of early diagnosis and improved neonatal intensive care and anesthesia. At present, in most of the developed countries, only the presence of associated major congenital anomalies determines the chances of survival.[3] This is not the same in developing countries, where many other preoperative, postoperative and socioeconomic factors continue to contribute to the persisting high mortality.[4,5] In the present study, we evaluated these prognostic factors that contribute to high mortality.

## MATERIALS AND METHODS

From February 2004 to May 2006, 127 neonates were admitted with the diagnosis of EA. The preoperative assessment of upper pouch was done with plain X-ray chest (posterior-anterior and lateral view) with 8 Fr Red Rubber catheter. The diagnosis of associated congenital anomalies was performed on the basis of careful systemic examination, radiological and sonological investigations. Data collected included age at the time of admission, gestational age, birth weight, sex, home/hospital delivery, history of feeding, associated congenital anomalies, respiratory status, presence of pneumonitis, type of anomaly, operative technique, gap (actual measurement done intraoperatively), complications and esophageal anastomotic leak (incidence, diagnosis and treatment) and their impact on survival. Waterston prognostic criteria were used for survival. We defined survival as an infant who leaves hospital and who is able to effectively take the feeds. The standard approach to EA was directed toward primary repair in all cases whenever possible except in cases of long gap, very low general condition or associated major anomalies. Surgery was performed with a retropleural approach with or without azygos ligation, retropleural drainage and transanastomotic stenting. TEF was ligated by ‘U’ transfixation with monofilament nonabsorbable suture. In almost all the cases, end-to-end esophageal anastomosis was performed by 5-0-polyglactin single layer interrupted sutures. All surgeries were performed using general anesthesia and patients were extubated right away postoperatively unless they had respiratory distress, associated cardiac anomalies or marked tension at the anastomotic site. If the patient was on an endotracheal tube, he or she was shifted to neonatal intensive care unit for ventilatory support. If the ventilator was not available, the patient was placed on ambu bag ventilation. If transanstomotic stenting was done, feeding was started after 24 h of surgery and gradually increased. If there was no evidence of any postoperative complications, contrast esophagogram was done on the seventh day of surgery. In patients who had a retropleural drain, anastomotic leaks after the primary repair were detected either by observing the saliva in the retropleural drain or by contrast study of the esophagus. Minor leaks were identified by appearance of frothy saliva in the retropleural drain with no accompanying deterioration in the general condition. An alternative method to confirm this was by giving oral methylene blue and then observing its appearance in the retropleural drain. Major leaks were clinically suspected by the contents draining with the accompanying deterioration in the general condition of the patient either due to mediastinitis or pneumonitis and septicemia. In patients who had no retropleural drain, leaks were clinically suspected by increased respiratory distress, fever and sepsis or plain X-ray chest showing pneumothorax and pneumonitis and confirmed by the contrast study of esophagus. The findings suggestive of leak were extravasation of contrast from esophagus into mediastinum. The finding of contrast in stomach without any clinical deterioration was considered normal.

The term minor leak was used for a small amount of extrapleural leakage and/or a small radiological leak and major leak referred to a large amount of drainage or a leak that caused respiratory symptoms associated with a large defect in anastomosis. Chi-square test and student's t- test was used for statistical analysis. *P* value < 0.001 was considered to be significant.

## RESULTS

Preoperative demographic details and their impact on survival are given in Table 1. Esophageal atresia with distal TEF was the commonest type present in 117 (92%) cases; pure EA was found in 9 (7%) cases and one case of EA was found with proximal and distal fistula. Associated congenital anomalies were present in 52 (41%) patients, including cardiac diseases in 17 patients, gastrointestinal in 15 cases, vertebral and nervous system anomalies in 8 cases, musculoskeletal anomalies in 6 cases, head and neck problems in 2 cases, genitourinary anomalies in 2 cases and respiratory system anomalies in 2 cases with cleft lip in 1 case. VACTERL association was present in 6 (5%) cases. Only 12 (9%) were having no respiratory distress at the time of admission; 44 (35%) were having mild, 58 (46%) were having moderate and 13(10%) were having severe respiratory distress. Clinically and/or radiologically chest was normal only in 17 (13%) cases, with mild pneumonitis in 51 (40%), moderate pneumonitis in 46 (40%) and severe pneumonitis in 13 (10%) cases. Short gap (<1 cm or one vertebral body) was found in 54 cases (50%), intermediate gap (1-3 cm or 1-3 vertebral bodies) in 36 (34%) and long gap (>3 cm or 3 vertebral bodies) in 17 (16%) cases. Survival rates were 89%, 60% and 23% in cases of mild, moderate and severe respiratory distress, respectively. Survival was 82% in patients with no preoperative pneumonitis falling down to 75%, 72% and 23% with mild pneumonitis, moderate pneumonitis and severe pneumonitis, respectively. Gap was a highly significant factor affecting the survival with 91% survival in short gap and dropping down to 69% and 53% in the intermediate gap and long gap, respectively. Survival according to Waterston criteria is shown in Table 2.

**Table 1:** Demographic preoperative details and their impact on survival (n=127)

Age at the time of admission		Total cases (%)	Survival (%)
	<24 h	46(36)	32 (70)
	24-48 h	34 (27)	24 (71)
	>48 h	47(37)	32 (68)
Maturity	Full term	88 (69)	70 (80)
	Preterm	39 (31)	18 (54)
Weight	>2.5 kg	60(47)	49 (82)
	1.8-2.5 kg	58 (46)	37 (64)
	<1.8 kg	9 (7)	2 (22)
Sex	Male	84 (66)	54 (64)
	Female	43 (34)	34 (79)
Place of delivery	Home	61 (48)	42 (69)
	Hospital	66 (52)	46 (70)
History of feeding	Present	70 (55)	48 (69)
	Absent	57 (46)	40 (70)

**Table 2:** Survival in patients with esophageal atresia in our series based on Waterston classification (*n* = 127)

Waterston classification	Total cases	Survival
		
	Number (%)	Number (%)
A	30 (24)	30 (100)
B	60 (47)	50 (83)
C	37 (29)	8 (22)

X^2^ = 58.369; *P* < 0.001

Primary definitive repair were performed in 99 cases of EA with or without TEF as given in Table 3. Our primary approach in all the patients of EA with TEF was extrapleural; however, in 7 cases of EA with TEF, extrapleural approach was converted to transpleural because of the severe inflammation of parietal pleura in patients with severe pneumonitis or inadvertent breeches in the pleura during the surgery.

**Table 3:** Survival related to operative procedure in esophageal atresia

	Preoperative mortality	Primary procedure		Staged procedure	
		
		Number	Survival (%)	Number	Survival (%)
EA with distal TEF (*n* = 117)	8	96	77 (80)	13	5 (38)
Pure EA (*n* = 9)	1	2	1 (50)	6	5 (83)
EA with proximal and distal TEF (*n* = 1)	0	1	0	0	-

In the primary repair of EA, azygos vein was preserved in 46 cases and retropleural drainage was not performed in 27 cases. Transanastomotic stenting for early feeding after 24 h of surgery was carried out in 53 (54%) cases. Cervical mobilization with proximal esophageal circular myotomy was conducted in one case and cervical mobilization alone was done in one case; Livaditis myotomy was done in 3 cases in EA with distal TEF. Abdominal mobilization of stomach and end-to-end esophageal anastomosis was done in 2 cases of pure EA. Staged procedures were done in 19 cases of EA as given in Table 3. Only one major leak was observed in the azygos-vein-preserved group as compared to azygos-vein-ligating group (*P* = < 0.001). Survival rate with retropleural drainage was 78% and without retropleural drainage was 85%. Anesthetic complications were faced in 11 cases; two patients had hypoxia and cardiac arrest. Intubation was difficult in 9 cases leading to tracheal injury, and out of these, 2 cases survived. Out of 118 cases of EA, 49 (42%) faced early postoperative complications; commonest complication was sepsis with pneumonitis, followed by sepsis alone. The commonest cause for mortality in cases with early postoperative complications was cardiorespiratory arrest secondary to hypoxia and pneumonitis in 11 cases. Delayed postoperative complications such as pneumonitis, sepsis, major anastomotic leak, aspiration and tracheomalacia were present in 51 (43%) cases that were responsible for mortality in 19 cases. Major anastomotic leak in patients of EA after primary repair was seen in 9/99 (9%). Management details are given in Table 4, and the incidence of minor leak was observed in 4 (4%) cases. In the delayed postoperative phase, seven patients expired because of major anastomotic leak and associated pneumonitis and septicemia.

**Table 4:** Management of major anastomotic leak in esophageal atresia

Management procedure	Number of cases	Cases survived
**EA with distal TEF (*n* = 8)**		
Conservative because of very low general condition	5	0
Cervical esophagostomy + gastrostomy + feeding jejunostomy	3	2
**Pure EA (*n* = 1)**		
Re-Right lateral thoracotomy + lavage of Rt. pleural cavity + gastric pull-up + reanastomosis	1	0

## DISCUSSION

The estimated incidence of EA in India is 18000 per year, and it is believed that only 10% of these babies reach a tertiary care centre.[6] Most tertiary care centers are situated in metropolitan cities and are ill-equipped in comparison to the western standard with respect to both manpower and machinery.[7] This study has led to several significant and useful observations and will provide us clues to achieve better results in future. The age at the time of admission is not a bad prognostic factor. Although none of the previous studies from abroad has considered age as a probable risk factor, for our study, it was necessary from the viewpoint that most of the patients were from centers situated far away and it took even days before they reached our hospital. The low survival rate amongst preterm does not indicate the failure of operative technique used; rather, it is a result owing to multitude of factors. Prematurity is still a major problem for developing countries due to the additional physiological handicaps in these babies and the increased susceptibility to sepsis.[7] Birth weight <2.5 kg is considered as a high risk factor and babies with birth weight <1.8 kg have the lowest survival rate because of the lack of advanced neonatological backup in our setup. Spitz *et al.*[8] *per se* did not regard weight as a contraindication for primary repair. Most of the studies reviewed are from developed countries and have not considered the place of delivery as a factor as most of the deliveries (particularly high-risk ones) are conducted in hospitals. Although our hospital serves as a referral centre to the state and serves the low socioeconomic classes, yet it showed a good survival rate as that in many well-equipped centers in rest of the world. The reason could be that the most of the cases among home delivery neonates came from far-off places, and only those cases that had high chances of survival reached our centre; the remaining cases might have died before reaching the hospital. This is because such neonates are first seen by persons other than the pediatric surgeon leading to the delay in diagnosis and increased mortality. Male to female ratio is 1.95:1 in our series, but it is a misinterpretation that EA is common in males. In most series, sex incidence is almost nearly equal. The fewer number of female enrolments could be attributed to low importance being given to female child in our society. It is usually seen that a daughter, that too, with congenital illness, is not welcome in a society like ours. Our findings are similar to that of Bindi *et al*.[9] The reason of absence of type B and only 1 case of type D in contrast to the studies from developed countries may be that deliveries there are conducted in hospitals and such cases are diagnosed early, thereby helping them to reach the desired centers in time. Associated anomalies were found in 52 (41%) cases and VACTERL association in 6 (5%) cases. These observations are not so different from those of Hassab *et al.*[10] who reported 60% associated anomalies with VACTERL association in 6%. Spitz *et al.*[8] reported 47%, Saing *et al.*[11] reported 59% and Rokitansky *et al.*[12] reported 52.4% associated congenital anomalies. The survival rate among these cases was low (43%) as compared with 78% in those free from any other congenital anomaly. This shows that association of other congenital anomalies plays a major role in the survival of patients with EA (X^2^ = 19.497; *P* < 0.001). The survival rate among the patients with EA with congenital heart disease (CHD) was 33% (*P* < 0.001), while in the series of Ein *et al.*,[13] 64% of the neonates of EA with CHD survived. Similar findings had been reported by Choudhury *et al*.[14] GI anomalies were responsible for mortality in 4 out of 39 cases (10%), while in series of Andrassy *et al.,*[15] these were responsible for mortality in 33% cases. The presence of long gap is significantly associated with the poor survival rate (*P* < 0.001). This is because the long gap is associated with high incidence of anastomotic complication and other congenital malformations. Brown *et al.*[16] and Sharma *et al.*,[17] in their study of the measurement of the gap length and mortality in EA, also reached the same conclusions. Long-gap atresia is a problem as well as a challenge to the pediatric surgeons because they require modifications from the conventional operation. Respiratory distress has an established association with mortality (*P* < 0.001). A significant association between the severity of pneumonitis and mortality was observed (*P* = < 0.001). Severe pneumonitis is because of the misguided efforts at diagnosis and at times the performance of oral contrast studies lead to a high incidence of severe aspiration chemical pneumonitis. Anesthetic complications were faced in 11 cases; this indicates that the anesthetist should be more competent and have sufficient exposure in handling the neonatal intubation and should be aware of the associated life-threatening anatomical anomalies (particulary, cardiac and anterior placement of larynx). The history of feeding that is present in several patients in our study is because of illiteracy and a tradition of giving tea after the birth of a neonate, particulary in the eastern part of Uttar Pradesh and the misdiagnosis, even after the appearance of the clinical symptoms. The survival rate was statistically not significant; the reason may be that the neonates who were fed did not have life-threatening anomalies. Sparing the azygos vein is not universal, but this has been described in the literature. Our hypothesis regarding low incidence of major anastomotic leak in azygos-preserved group is that preservation of the azygos vein allows normal venous drainage from the esophagus, thereby precluding the edema at the anastomotic site and protecting against anastomotic disruption. No recurrent TEF were found in our series. Transanastomotic stenting and early feeding makes no significant difference in survival. Kevin *et al,*[18] also concluded that transanastomotic feeding tubes and early enteral nutrition are safe and cost effective. Retropleural drainage was performed only in patients with moderate to severe gaps in which anastomosis was under tension. Gangopadhyay *et al.*[19] also recommended that retropleural drainage is not necessary in all the cases of EA.

In the present study, 96/117 (82%) patients of EA with TEF had primary repair in which 77(80%) patients survived as compared with 36% survival rate in the series of Sarin *et al*.[19] and 35-50% in other studies conducted in India.[20,21] Long gap or low general condition forced us for staged procedures (cervical esophagostomy and abdominal esophagostomy or cervical esophagostomy and ligation of the distal esophageal end with gastrostomy and feeding jejunostomy). Only 5 patients out of 13 survived (38%). Bhatnagar *et al*.[22] studied the exteriorization of the distal esophagus in the abdomen in EA patients with indications of long-gap atresia with or without TEF. Out of 118 cases of EA, 47(40%) faced early postoperative complications that were responsible for mortality in 11 cases. The most common complication was sepsis alone or sepsis with pneumonitis or meningitis; however, the most common cause for mortality was cardiorespiratory arrest (may be due to associated life-threatening anomalies, severe pneumonitis and hypothermia *vs* hyperthermia, hypoglycemia and anesthetic problems). Most of these early postoperative complications are not related with surgical procedures. Delayed postoperative complications such as pneumonitis, sepsis, major anastomotic leak, aspiration and tracheomalacia were present in 51 (43%) cases, and these complications were responsible for mortality in 19 cases. Factors predicting mortality were pneumonia and sepsis at presentation or that acquired during hospitalization, major or life-threatening anomalies, long gaps and major leaks. Similar postoperative complications are also reported by Bindi *et al.*[9] and Hassab *et al.*[10] The incidence of anastomotic leak in patients of EA after primary repair was observed in 13 patients with major leak in 9 (9%) and minor leak in 4 (4%) patients. Spitz *et al.*[8] and McKinnon and Kosloske *et al.*[23] also reported anastomotic leak in 21% cases. Amongst patients with major leak, seven patients associated with pneumonitis or septicemia expired. On comparing the survival rate among patients with major leak and patients without major leak the difference was found to be statistically significant (X^2^ = 18.953; *p* < 0.001). Sarin *et al.*[24] reported poor results with anastomotic leaks with only 20% survival rate amongst the leak group. Statistically significant difference was observed between the survival rates among different classes of Waterston.

Spitz *et al.*[8] had earlier reported that survival rates according to Waterston classification was 100% for class A, 86% for class B and 73% for class C cases. In the series of Bhatnagar *et al.*,[7] the survival was maximum in group A (67.6%) and it dropped down to 28.8% in group C. At present, the survival rate has improved in Group A and B; however, it has remained almost the same in group C. On comparing the data of the present series with that of Hassab *et al.,*[10] it was established that although the distribution of cases as per Waterston classification in both these setups were different, the survival rates were almost similar. Our results for survival in class C are lower as compared with other studies, for which the reason might be the higher incidence of low birth weight, delayed diagnosis, poor unsupervised transport, low socioeconomic status and lack of advanced neonatological back up. Waterston classification was statistically the best application in our study. We also propose that the survival in EA can be used as an index for the status of neonatal surgical care because EA had the highest mortality rate amongst all the surgical conditions because of the problems in respiratory care and surgical technical failure.[25]
